# Microbiological Laboratory Diagnosis of Human Brucellosis: An Overview

**DOI:** 10.3390/pathogens10121623

**Published:** 2021-12-14

**Authors:** Giovanni Di Bonaventura, Silvia Angeletti, Andrea Ianni, Tommasangelo Petitti, Giovanni Gherardi

**Affiliations:** 1 Department of Medical, Oral and Biotechnological Sciences, “G. d’Annunzio” University of Chieti-Pescara, Via Luigi Polacchi 11, 66100 Chieti, Italy; gdibonaventura@unich.it; 2 Center for Advanced Studies and Technology (CAST), “G. d’Annunzio” University of Chieti-Pescara, Via Luigi Polacchi 11, 66100 Chieti, Italy; 3 Department of Medicine, Campus Biomedico University, Via Alvaro del Portillo 200, 00128 Rome, Italy; s.angeletti@unicampus.it (S.A.); a.ianni@unicampus.it (A.I.); t.petitti@unicampus.it (T.P.)

**Keywords:** brucellosis, laboratory diagnosis, culture method, serological test, nucleic acid amplification test

## Abstract

*Brucella* spp. are Gram-negative, non-motile, non-spore-forming, slow-growing, facultative intracellular bacteria causing brucellosis. Brucellosis is an endemic of specific geographic areas and, although underreported, represents the most common zoonotic infection, with an annual global incidence of 500,000 cases among humans. Humans represent an occasional host where the infection is mainly caused by *B. melitensis*, which is the most virulent; *B. abortus*; *B. suis*; and *B. canis*. A microbiological analysis is crucial to identifying human cases because clinical symptoms of human brucellosis are variable and aspecific. The laboratory diagnosis is based on three different microbiological approaches: (i) direct diagnosis by culture, (ii) indirect diagnosis by serological tests, and (iii) direct rapid diagnosis by molecular PCR-based methods. Despite the established experience with serological tests and highly sensitive nucleic acid amplification tests (NAATs), a culture is still considered the “gold standard” in the laboratory diagnosis of brucellosis due to its clinical and epidemiological relevance. Moreover, the automated BC systems now available have increased the sensitivity of BCs and shortened the time to detection of *Brucella* species. The main limitations of serological tests are the lack of common interpretative criteria, the suboptimal specificity due to interspecies cross-reactivity, and the low sensitivity during the early stage of disease. Despite that, serological tests remain the main diagnostic tool, especially in endemic areas because they are inexpensive, user friendly, and have high negative predictive value. Promising serological tests based on new synthetic antigens have been recently developed together with novel point-of-care tests without the need for dedicated equipment and expertise. NAATs are rapid tests that can help diagnose brucellosis in a few hours with high sensitivity and specificity. Nevertheless, the interpretation of NAAT-positive results requires attention because it may not necessarily indicate an active infection but rather a low bacterial inoculum, DNA from dead bacteria, or a patient that has recovered. Refined NAATs should be developed, and their performances should be compared with those of commercial and home-made molecular tests before being commercialized for the diagnosis of brucellosis. Here, we review and report the most common and updated microbiological diagnostic methods currently available for the laboratory diagnosis of brucellosis.

## 1. Laboratory Diagnosis of *Brucella* Infection

*Brucella* spp. are small (0.5–0.7 by 0.6–1.5 µm), non-motile, non-spore-forming, slow-growing, facultative intracellular Gram-negative coccobacilli belonging to the *Brucellaceae* family along with the *Mycoplana* and *Ochrobactrum* spp. [1,2]. Brucellae can infect several animal species and currently comprise 12 well-classified species, 4 of which causing almost the totality of human infections: *B. melitensis*, *B. abortus*, *B. suis*, and *B. canis*, with *B. melitensis* being the most virulent [3]. DNA-DNA hybridization studies have demonstrated that the different *Brucella* species are very closely related, with a percentage of similarity close to 100%, and they can be considered different subspecies belonging to a single species [4]. Nevertheless, the sequencing of several specific genes indicated that specific genotypes are strongly correlated with the different species, supporting the usefulness of retaining the traditional classification. Moreover, the conventional nomenclature has been maintained because of clinical, practical, and epidemiological reasons, with different biovars being species specific, such as *B. abortus* being closely associated with cattle, *B. melitensis* being closely associated with small ruminants, *B. suis* being closely associated with swine, and *B. canis* being closely associated with canids.

Brucellosis is a zoonotic infection due to the ability of these species to infect non-preferential hosts, including humans [3]. It can affect any organ and body site, occurring in animals and humans, as an occasional host, with very rare cases of human-to-human transmission [5]. Endemic in some geographic areas, such as the eastern Mediterranean basin, the Middle East, Arabian Peninsula, Latin America, Southern Europe, Central Asia, the Indian subcontinent, and many African countries, brucellosis represents the most common zoonotic infection, with a total of about 500,000 new human cases per year. However, a high discrepancy between the reported rate and the actual incidence has been reported largely due to misdiagnosis and underdiagnosis, especially in endemic areas [6]. The One Health approach, based on the integration of human and animal health, plants, and ecosystems, which involves local, regional, national, and global multidisciplinary cooperation and efforts, is useful [7].

The treatment of brucellosis is a challenge for many physicians since it requires prolonged therapy with a combination of antimicrobial drugs rarely used for other types of bacterial infections [8,9]. The fast and precise diagnosis of human brucellosis is essential for the delivery of a prompt and adequate antimicrobial therapy. It also supports public health services by identifying exposure to sick animals and avoiding the consumption of contaminated food. 

**Figure 1:**
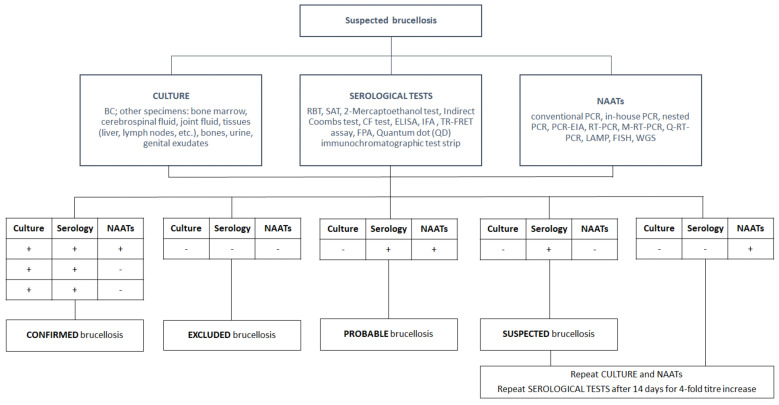
Diagnostic algorithm for human brucellosis.

Due to the variable and nonspecific clinical symptoms in humans, the microbiological laboratory is crucial for the identification of human cases and their subsequent management. A laboratory diagnosis can be carried out using three different approaches and microbiological procedures: direct diagnosis by culture, indirect diagnosis by serological tests, and rapid diagnosis by molecular PCR-based methods. The main features of the most common diagnostic approaches herein reviewed are summarized in Table 1, and the diagnostic algorithm for human brucellosis is depicted in Figure 1.

## 2. Culture

A culture is considered the “gold standard” for the laboratory diagnosis of brucellosis. As human brucellosis pathogenesis is always characterized by an initial bacteremic phase, peripheral blood cultures (BC) should always be performed as soon as brucellosis is suspected. This represents an important method to confirm the disease, although it shows a sensitivity ranging from 10 and 90% [9]. The variables that could affect the positivity rate of BC are microbial (i.e., *Brucella* species involved), patient-related (e.g., age, duration of symptoms, acute or chronic and focal disease, first infection or relapse, and antibiotics use), and method-associated (e.g., blood volume, number of BC vials collected, BC system used, incubation time, and performance of blinded subcultures) [10]. BC isolation of *Brucella* spp. with accurate identification to the species level allows for defining the source of infection and the initial stage of the disease when serological tests are still negative or inconclusive and for differentiating between wild-type and vaccine *Brucella* strains [11,12]. During the initial stage of brucellosis, patients present a low bacterial load in the blood that can be only detected by following the recommended guidelines of BC collection, i.e., drawing at least two or three separate BC sets and avoiding the collection of only a single BC set (“solitary” BC) [13]. As the infection progresses, the organism is removed from the blood and enters the macrophages, with a reduction in the concentration of circulating bacteria, which makes their isolation difficult [14].

The characteristics of slow growth (i.e., several hours), low bacterial load in blood, and CO_2_ production of brucellae, require a modification of the common procedures used in the automated BC systems [15,16]. Although the normal time of incubation in the automatic BC systems is up to 5 days, the incubation needs to be prolonged up to 7 days to detect severe cases of brucellosis [17,18]. In some cases, the American Society for Microbiology and the WHO recommend prolonging the incubation of BC bottles up to 4 weeks along with blinded subcultures, although this protocol presents several disadvantages, such as expensive and labor-intensive work, organizational problems, and delay in the diagnosis from automatic BC systems [10,19].

Different BC methods have been used for the detection of *Brucella* spp., such as the manual monophasic and biphasic methods [20], lysis-based BC (white blood cells are lysed by saponin to release *Brucella* prior to inoculate them onto culture media), blood clot cultures (blood clots in which leukocytes containing phagocytosed organisms are cultured), and the automated new generation BC systems, with the most common being the Bactec 9000 or Bactec FX series from Becton Dickinson and the BacT/Alert BC system from bioMerieux. The use of new BC instruments has increased the sensitivity of blood cultures and shortened the time to detection of *Brucella* species [21,22].

After the initial hematogenous spread, in 25–35% of patients, brucellae can move towards remote organs with the development of focal infections. This allows for the isolation of brucellae from specimens in other sites other than blood, such as bone marrow, urines, bone tissue, pleural and synovial fluid aspirates, liver biopsy specimens, lymph nodes, cerebrospinal fluid, and abscesses after an incubation up to 14 days in a 5% CO_2_-enriched atmosphere at 35 °C or by inoculation of specimens into broth media (usually those used for blood cultures) [23,24,25,26,27,28,29,30].

The rapid identification of *Brucella* spp. recovered from culture is essential (i) to establish a timely diagnosis; (ii) to avoid biological risk for laboratory personnel; (iii) to confirm the presence of the disease in its early stages, when antibody titers are still absent, low, or borderline; (iv) to distinguish between wild and vaccine *Brucella* strains; and (v) to track the source, since the individual species and their naturally occurring hosts are strongly associated.

Clinical microbiology laboratories should identify suspected colonies based on few morphologic, biochemical, and serological tests. *Brucella* spp. can be isolated mostly from blood agar and chocolate agar media after 24–72 h of incubation. Colonies are small (1–2 mm), convex, nonpigmented, nonhemolytic with an entire edge, and frequently smooth. Rough variants can occur with *B. canis* [31]. The presumptive identification of brucellae is based on the Gram staining appearance (faintly stained Gram-negative very small coccobacilli resembling fine sand, often clustered in microcolonies); specific traditional phenotypic reactions, such as positive oxidase, catalase, and urease activity; no fermentation of sugars; H_2_S production; growth in the presence of thionine and basic fuchsin; oxidative metabolic tests; and lack of motility.

Several commercial systems may misidentify brucellae for closely related bacterial species, mostly belonging to the related genus *Ochrobactrum* [75]. Besides conventional phenotypic methods, the identification of brucellae to the species level can be also performed by Matrix-Assisted Laser Desorption Ionization–Time Of Flight mass spectrometry (MALDI–TOF) technology, although its accuracy is sometimes contradictory [76,77,78,79]. The presumptive identification of brucellae should be confirmed by a molecular method.

Brucellae represent the most common etiology of laboratory-acquired infections [80] because of the high transmission of these microorganisms within confined environments. Indeed, a very low infectious dose (10–100 cells by aerosol or subcutaneous routes) is required, and several entry routes to access the host are exploited by these bacteria (e.g., respiratory mucosa, conjunctivae, abraded skin, and gastrointestinal tract) [80]. In addition, viable brucellae can persist in the environment for weeks and even months [80].

Routine bacteriological manual procedures such as homogenization of tissues, centrifugation and vortexing of bacterial suspensions, performance of subcultures, and biochemical testing may create dangerous aerosols and spillage of living bacteria, increasing the risk for unintentional transmission to working personnel [81].

The Centers for Disease Control recommended BSL-2 practices, containment equipment, and facilities for routine handling of clinical specimens of human or animal origins, whereas BSL-3 practices, containment equipment, and facilities are recommended for all manipulations of cultures of pathogenic *Brucella* spp. [82]. Furthermore, in areas of endemicity of brucellosis, all positive blood culture vials should be initially processed in safety cabinets, pending final identification of the organism. Bacterial isolates presumptively identified as *Bordetella bronchiseptica, Ochrobactrum* spp., *Bergeyella zoohelcum*, or *Psychrobacter phenylpyruvicus* should be managed in a similar manner until the presence of a *Brucella* spp. has been firmly ruled out.

## 3. Serological Tests

Indirect diagnosis is based on serological tests aimed at detecting specific antibodies in the serum of patients. Interpretive criteria are uniformly defined, such as a highly specific titer in agglutination assays, a cutoff value in enzyme-linked immunosorbent assay (ELISA), or the presence of a clear specific band in the lateral flow immunoassay. Nevertheless, these criteria are often controversial depending on laboratory-dependent differences and on the clinical and epidemiological characteristics (e.g., age, duration of illness, occupational risks, history of the disease, endemicity, and repeated exposition) [83,84,85]. The main disadvantages of serological tests are their low specificity; the difficulty in interpretating results, especially in patients with repeated exposure to *Brucella*; and distinguishing between active and past infection [33]. Serology showed also low sensitivity during the early stage of the disease and suboptimal specificity due to cross-reaction with other bacterial species. Other major causes of false-negative results of serological tests are prozone effect, low-affinity antibodies, and infections due to *B. canis* [10]. Despite these limitations, serological tests represent the main diagnostic methods for the diagnosis of brucellosis in endemic and low-to-middle-income countries because they are low cost and user friendly and have a high negative predictive value.

A wide variety of serological tests for the diagnosis of human brucellosis are available, mostly applied in the veterinary field. The major challenge for the development of serological tests is the complexity of antigenic structures, such as outer membrane proteins, cytosolic proteins, and immunodominant LPS. Several antigens are used for serological tests, mostly obtained from *B. melitensis* and *B. abortus*, whereas whole cell preparations are used in the indirect fluorescent-antibody (IFA) [31]. However, most serological tests used for the laboratory diagnosis of brucellosis are divided into methods that target brucellar smooth LPS (S-LPS) and those targeting cytosolic proteins.

The methods directed at S-LPS comprise the Rose Bengal test (RBT), based on a slide agglutination test [32]; the standard agglutination test (SAT) in tubes, the most common serological assay used for diagnosing *B. abortus*, *B. melitensis*, and *B. suis* infections, with the new developed SAT miniaturized test [6,12,33,34]; the 2-ME test, which uses 2-mercaptoethanol to eliminate the IgM type, leaving only the IgG isotype [35]; the Coombs antiglobulin agglutination test [36] and the *Brucella* Coombs gel test [37,38]; the complement fixation test (CF) [39]; the immunocapture agglutination test, such as BrucellaCap [40,41]; the IgG avidity ELISA [42]; the fluorescent resonance energy transfer (FRET) assay, which labels a given antigen and its complementary antibody with adequate fluorophores and measures the amount of energy transferred after the excitation of the donor fluorophore [44] against the *Brucella* S-LPS; and the fluorescent polarization immunoassay (FPA), based on measuring the difference in rotational velocity between a small antigen molecule in solution, labeled with a fluorochrome, and the same antigen conjugated with its antibody, against brucellar S-LPS [45].

The major test targeting cytosolic proteins is ELISA, also used directly on cerebrospinal fluid (CSF) samples for neurobrucellosis diagnosis [6,43]. Recently, rapid detection of brucellosis using a handheld quantum dot (QD) immunochromatographic test strip can be employed as preliminary screening of brucellosis [46]. Contrary to enzyme immunoassay (EIA) and IFA, agglutination-based tests cannot differentiate the types of antibodies involved [1]. Novel experimental antigens and tests have been developed, i.e., synthetic oligosaccharides, recombinant *Brucella* proteins-providing higher sensitivity, rapid and reliable results, reduction in costs, and simplicity of technical execution [86,87,88,89,90].

## 4. Nucleic Acid Amplification Tests (NAATs)

Molecular methods, also called NAATs, allow for the diagnosis of brucellosis in a few hours with high sensitivity and specificity. NAATs remain positive for a long time in patients apparently asymptomatic and when clinical relevance is unclear. However, a positive test may not necessarily indicate an active infection but could be the result of a low bacterial inoculum in frequently exposed healthy individuals, DNA from dead organisms, or successfully treated patients. Thus, the interpretation of results from NAATs should be carefully conducted, always taking into consideration the clinical and epidemiological setting involved.

Used first on peripheral blood with good performance, serum samples represent the sample of choice for the molecular diagnosis of human brucellosis with better yield [47,48,49]. A molecular diagnosis of brucellosis can be also performed in other specimens (e.g., from the genitourinary, osteoarticular, cardiovascular, and central nervous systems), useful in the diagnosis of focal brucellosis affecting any organ and tissue where cultures are often negative [91,92]. Moreover, Formalin-Fixed Paraffin-Embedded (FFPE) tissue acquired from surgical biopsy samples can be used following validated DNA extraction procedures [50].

The specific targets used for molecular tests involve mainly genes encoding outer membrane proteins, such as *omp2* and *omp31*, and *omp28* genes, also named *bp26* [51,52,53,54]. Other gene targets used for molecular diagnosis of *Brucella* infection are [55,56,57,58] (i) the gene 16S rRNA, although cross-reactions have been reported; (ii) the insertion sequence IS*711*, in which the performance has been questioned due to its sequence variation and its absence in some strains; and (iii) *bcsp31*, the most frequently used gene, encoding the synthesis of an immunogenic membrane protein.

Amplification strategies in NAATs include conventional PCR methods, in-house PCR, nested PCR, PCR-enzyme immunoassay (PCR-EIA) in a microplate format, real-time PCR, quantitative RT-PCR, and multiplex real-time PCR (M-RT-PCR) [10,59,60,61,62,63,64]. Recently, a new method based on loop-mediated isothermal amplification (LAMP) has been developed for the detection of brucellae [65].

Routine performance of antibiotic susceptibility tests is not recommended for *Brucella* isolates due to their susceptibilities to first-line antimicrobial agents [93]. However, it is recommended that all strains should be sent to a reference laboratory for accurate identification to the species level and for determination of its biovar, for several reasons, such as the identification of zoonotic source, epidemiological studies during outbreaks, the description of strains circulating and spreading in a particular geographic area, the differentiation between wild-type and vaccine strains, and veterinary control programs [10,94].

Molecular methods used to identify brucellae to species level and genotyping include (i) fluorescence in situ hybridization (FISH) test, targeting a partial region of the 16S rRNA gene with rapid and precise detection of all human pathogenic species; (ii) a novel *recA* gene-based PCR assay able to differentiate between *Brucella* spp. and the related species *Ochrobactrum* spp.; (iii) the “AMOS PCR” test used to differentiate four *Brucella* species, namely *B. abortus*, *B. melitensis*, *B. ovis*, and *B. suis* and vaccine strains; (iv) the Bruce ladder multiplex PCR assay, proving to identify and accurately differentiate between reference and vaccine isolates with high reproducibility; and (v) whole genome sequencing by the identification of specific SNPs with the differentiation of five different *Brucella* species [66,67,68,69,70,71].

Commercial NAATs available for the diagnosis of brucellosis are still limited, and published comparative studies for assessing the different performances of commercial and home-made molecular tests are scarce and, in several cases, only report a small sample size [72,73]. Rapid, reliable, and affordable detection of *Brucella* spp. via molecular methods remains a challenge. Thus far, there are not validated commercial or home-made NAATs that can guarantee a high reproducibility of results; thus, direct methods by culture and indirect methods by serological tests remain the main tools for the laboratory diagnosis of brucellosis and the methods of choice for the follow-up of infections by *Brucella* spp.

## 5. Conclusions

In this mini review, we reported and discussed the most updated and common methods currently available for the microbiological laboratory diagnosis of brucellosis. The fast and precise diagnosis of human brucellosis is essential for delivering a prompt and adequate antimicrobial therapy, for informing public health services, and for avoiding the spread of the disease through exposure to sick animals and the consumption of contaminated food. Moreover, the clinical symptoms of human brucellosis are variable and nonspecific; therefore, a microbiological laboratory analysis is essential.

A laboratory diagnosis is based on three different microbiological approaches: direct diagnosis by culture, indirect diagnosis by serological tests, and rapid diagnosis by molecular NAATs.

Despite the availability of long-term experience (serological) and highly sensitive (NAATs) tests, a culture is still considered the “gold standard” in the laboratory diagnosis of brucellosis due to its clinical and epidemiological relevance. The recent development of automated BC systems has increased the sensitivity of BCs and shortened the detection time of *Brucella* species by monitoring CO_2_ production, thus achieving a detection rate of more than 95% within a one week incubation period. Prolonged incubation is required in the case of focal complications along with blind subcultures. Rapid and accurate identification of *Brucella* species has been accomplished with the introduction of MALDI-TOF analysis, nucleic acid amplification assays, and hybridization tests.

The indirect diagnosis is based on serological tests detecting antibodies in the serum of patients. The main limitations of serological tests are the lack of common interpretative criteria (depending on laboratory variability, and clinical and epidemiological differences), the suboptimal specificity due to interspecies cross-reactivity, and the low sensitivity during the early stage of disease. Despite being imperfect, serological tests remain the main diagnostic tool in endemic areas thanks to their affordability, user friendliness, and high negative predictive value. Generally, a sensitive serological test method is used as an initial screening followed by a more specific confirmatory test such as the SAT. Despite the development of new serological tests being hampered by the complexity of antigenic structures, promising serological tests based on new synthetic antigens have been recently developed. In addition, a novel point-of-care test could warrant reliable results without the need for dedicated equipment and expertise, or the shipping of samples to distant laboratories.

NAATs are rapid tests that can help to diagnose brucellosis in a few hours with high sensitivity and specificity. Nevertheless, the interpretation of NAATs results requires attention because a positive test may not necessarily indicate an active infection but rather a low bacterial inoculum, DNA from dead organisms, or recovered patients. Commercial NAATs available for the diagnosis of brucellosis are still limited, and published comparative studies assessing the different performances of commercial and home-made molecular tests are still scarce.

The One Health approach, based on globally integrated multidisciplinary efforts on human and animal health, should be pursued for the optimal management of brucellosis.

## Figures and Tables

**Table 1:** Features of the common diagnostic tests (culture, serological, and molecular tests) used for laboratory diagnosis of brucellosis.

Diagnostic Approach	Diagnostic Test	Principle	Recommended Use	Advantages	Disadvantages	References	Sample Types	Diagnosis of Acute (A) or Chronic (C) Infections
Direct	Culture	Isolation from BC ^a^ and other specimens (bone marrow, cerebrospinal fluid, joint fluid, tissues (liver, lymph nodes, etc.), bones, urine, and genital exudates)	BC samples should be collected as soon as possible when BSI is suspected, even in the absence of fever. Drawing two or three separate BC sets due to a continuous low-grade bacteremia.	The positivity provides definitive diagnosis. Confirm the diagnosis of brucellosis in its early stages, when the serology is still negative or shows low/borderline antibody titers.	Slow growth. Variable yields highly dependent on BC instruments used. Needs biosafety cabinet.	[9,10,11,12,13,14,15,16,17,18,19,20,21,22,23,24,25,26,27,28,29,30,31]	BC, other specimens (bone marrow, cerebrospinal fluid, joint fluid, tissues (liver, lymph nodes, etc.), bones, urine, and genital exudates)	A
Indirect (serological tests)	Rose Bengal test (RBT)	Slide agglutination test detecting agglutinating and non-agglutinating antibodies.	Screening tool (positive result to be confirmed with SAT). Good accuracy in acute cases.	Fast (10 min) and simple. No drawback due to the prozone phenomenon.	Low sensitivity in complicated and chronic cases. False-positive results due to cross-reaction.	[32]	serum	A, C
	Tube standard agglutination test (SAT) or microplate agglutination	Detection of antibodies to brucellar S-LPS.	Widely used. Relatively good in acute cases.	Microagglutination tests require small amounts of reagents and low serum volumes and allow for simultaneous testing of multiple samples and results in a shortened turnaround time.	Not useful for *B. canis* infection. High rate of false negatives in complicated and chronic cases. False-positive results due to cross-reaction, non-agglutinating antibodies, and prozone effect. Takes time to set up and 24 h to read.	[6,12,33,34]	serum	A, C
	2-Mercaptoethanol test	Chemical inactivation of the agglutinating capabilities of the IgM pentamer by 2-mercaptoethanol.	Monitoring of the response to antimicrobial agents in already diagnosed patients; early detection of treatment failure.	Elimination of IgM confounder.	Turns positive later than SAT. Works in a fume hood.	[35]	serum	A, C
	Indirect Coombs test (Coombs antiglobulin agglutination test and *Brucella* Coombs gel test)	Extension of SAT. Detects non-agglutinating antibodies or incomplete antibodies.	Diagnosis of chronic infections and relapses. Detection of incomplete antibodies.	*Brucella* Coombs gel test is rapid and simple with results in 2 h, but only few studies are reported.	Time- and labor-consuming test that takes an additional 24 h to read.	[36,37,38]	serum	C
	Complement fixation (CF) test	Detection of IgG1 isotype antibodies by complement fixation.	Used in control/eradication programs for the serological diagnosis of the zoonosis in animals.		Not commonly used in human infection due to its technical complexity and problems in its standardization.	[39]	serum	A, C
	Immunocapture agglutination test (BrucellaCapt test; Vircell, Granada, Spain)	Detection, in a single step, of agglutinating IgG and IgM antibodies as well as non-agglutinating antibodies to the three smooth O-polysaccharide-containing *Brucella* spp.	Diagnosis confirmation. Follow-up of treated patients.	Performance comparable with that of Coombs test but it is more rapid and easier to carry out. Results read in 24 h.	Wide differences between individuals, and relapsed patients can exhibit a one-dilution decrease in the titer.	[40,41]	serum	A, C
	Enzymatic Linked Immuno Sorbent Assay (ELISA)	Plates are usually sensitized with cytosolic protein antigens. It can be also applied for the detection of S-LPS.	Test of choice for complicated, focal, and chronic cases. Diagnosis confirmation. Diagnosis of *B. canis* infection and neurobrucellosis.	When other tests are negative, detects total and individual specific Igs (IgG, IgM, and IgA). Rapid (4–6 h). Objective. Highly sensitive. Allows for simultaneous testing of multiple samples, (used for epidemiological serosurveys).	Less specific than agglutination test. False-negative results for anti-*Brucella* IgM antibodies. False-positive results due to the presence of rheumatoid factor.	[6,42,43]	serum	A, C
	Immuno-fluorescence assay (IFA)	Whole cell preparations as antigens.		Accuracy comparable to ELISA. Rapid (2–3 h).	It is subjective. May fail to detect IgA.	[31]	serum	A, C
	Time-resolved fluorescent resonance energy transfer (TR-FRET) assay	Uses antigens and antibodies labelled with fluorophores, based on energy transfer between them. Serum antibodies against the *Brucella* S-LPS outcompete a labeled monoclonal antibody that is also specific for this antigen.		Rapid (requires a single 30 min incubation time and no washing steps, followed by fluorescence read). Performance comparable with other methods.		[44]	serum	A, C
	Fluorescent polarization immunoassay (FPA)	Measures the difference in rotational velocity between a small antigen molecule in solution, labeled with a fluorochrome, and the same antigen molecule conjugated with its antibody.	Diagnosis of the zoonosis in domestic and feral animals as well as for brucellosis screening in the dairy industry.	Successfully used in animals. Only one study in human infections with high sensitivity and specificity. Rapid. Easy to perform. Portable equipment.		[45]	serum	A, C
	Quantum dot (QD) immunochromatographic test strip	Handheld QD immunochromatographic strip device.	Used as point-of-care testing for rapid detection and preliminary screening of brucellosis.	Fast. Easy to perform. Highly sensitive and specific, and comparable with SAT.		[46]	serum	A
Rapid (molecular tests)	NAATs ^b^ (conventional PCR, in-house PCR, nested PCR, PCR-EIA, RT-PCR, M-RT-PCR, Q-RT-PCR, LAMP, FISH, and WGS)	Serum is the sample of choice for NAAT-based diagnosis of human brucellosis.	Can be used for the diagnosis of brucellosis in human patients with focal complications.	Very highly sensitive (88–100%) and specific (92–100%).	Needed more comparative studies with culture and serology to introduce NAATs in clinical laboratory routines.	[47,48,49,50,51,52,53,54,55,56,57,58,59,60,61,62,63,64,65,66,67,68,69,70,71,72,73]	BC, serum, or other specimens for focal infections (bone marrow, cerebrospinal fluid, joint fluid, tissues (liver, lymph nodes, etc.), bones, urine, and genital exudates)	A, C ^c^

^a^ BC, blood culture. ^b^ NAATs: Nucleic acid amplification tests; PCR: polymerase chain reaction; PCR-EIA, PCR enzymatic immuno-assay; RT-PCR: real-time PCR; M-RT-PCR: multiplex real-time PCR; Q-RT-PCR: quantitative real-time PCR; LAMP: loop-mediated isothermal amplification; FISH: fluorescence in situ hybridization; WGS: whole genome sequencing. ^c^ Depending on the target genes used [74].

## Abbreviations

BC	blood culture
RBT	Rose Bengal test
SAT	standard agglutination test
CF	complement fixation
ELISA	enzyme-linked immunosorbent assay
IFA	indirect fluorescent-antibody
TR-FRET	time-resolved fluorescent resonance energy transfer
FPA	fluorescent polarization immunoassay
NAATs	nucleic acid amplification tests
PCR	polymerase chain reaction
PCR-EIA	PCR enzymatic immuno-assay
RT-PCR	real-time PCR
M-RT-PCR	multiplex real-time PCR
Q-RT-PCR	quantitative real-time PCR
LAMP	loop-mediated isothermal amplification
FISH	fluorescence in situ hybridization
WGS	whole genome sequencing

## References

[1] Araj G.F. (2019). Brucella. Manual for Clinical Microbiology.

[2] Jiao H., Zhou Z., Li B., Xiao Y., Li M., Zeng H., Guo X., Gu G. (2021). The mechanism of facultative intracellular parasitism of *Brucella*. Int. J. Mol. Sci..

[3] Whatmore A.M., Koylass M.S., Muchowski J., Edwards-Smallbone J., Gopaul K.K., Perrett L.L. (2016). Extended multilocus sequence analysis to describe the global population structure of the genus *Brucella: Phylogeography* and relationship to biovars. Front. Microbiol..

[4] Al Dahouk S., Scholz H.C., Tomaso H., Bahn P., Gollner C., Karges W., Appel B., Hensel A., Neubauer H., Nockler K. (2010). Differential phenotyping of *Brucella* species using a newly developed semi-automated metabolic system. BMC Microbiol..

[5] Mesner O., Riesenberg K., Biliar N., Borstein E., Bouhnik L., Peled N., Yagupsky P. (2007). The many faces of human-to-human transmission of brucellosis: Congenital infection and outbreak of nosocomial disease related to an unrecognized clinical case. Clin. Infect. Dis..

[6] Franco M.P., Mulder M., Gilman R.H., Smits H.L. (2007). Human brucellosis. Lancet Infect. Dis..

[7] Ghanbari M.K., Gorji H.A., Behzadifar M., Sanee N., Mehedi N., Bragazzi N.L. (2020). One health approach to tackle brucellosis: A systematic review. Trop. Med. Health.

[8] Bosilkovski M., Keramat F., Arapović J. (2021). The current therapeutical strategies in human brucellosis. Infection.

[9] Pappas G., Akritidis N., Bosilkovski M., Tsianos E. (2005). Brucellosis. N. Engl. J. Med..

[10] Yagupsky P., Morata P., Colmenero J.D. (2019). Laboratory diagnosis of human brucellosis. Clin. Microbiol. Rev..

[11] Tang L., Liu J., Wang Y., Zhang H., Chen C. (2017). Evaluation of a hypervariable octameric oligonucleotide fingerprints assay for identification of and discrimination between wild-type and vaccine strains of *Brucella melitensis*. Am. J. Vet. Res..

[12] Shemesh A.A., Yagupsky P. (2011). Limitations of the standard agglutination test for detecting patients with *Brucella melitensis* bacteremia. Vector Borne Zoonotic Dis..

[13] Baron E.J., Weinstein M.P., Dunne W.M., Yagupsky P., Welch D.F., Wilson D.M., Baron E.J. (2005). Blood Cultures IV. Cumitech 1C.

[14] Pappas G., Papadimitriou P. (2007). Challenges in *Brucella* bacteremia. Int. J. Antimicrob. Agents.

[15] Yagupsky P., Peled N., Press J., Abramson O., Abu-Rashid M. (1997). Comparison of BACTEC 9240 Peds Plus medium and Isolator 1.5 Microbial ube for detection of *Brucella melitensis* from blood cultures. J. Clin. Microbiol..

[16] Gamazo C., Vitas A.I., Lopez-Goni I., Diaz R., Moriyon I. (1993). Factors affecting detection of *Brucella melitensis* by BACTEC NR730, a nonradiometric system for hemocultures. J. Clin. Microbiol..

[17] Raj A., Gautam V., Gupta P.K., Sethi S., Rana S., Ray P. (2014). Rapid detection of *Brucella* by an automated blood culture system at a tertiary care hospital of north India. Indian J. Med. Res..

[18] Esel D., Doganay M., Alp E., Sumerkan B. (2003). Prospective evaluation of blood cultures in a Turkish university hospital: Epidemiology, microbiology and patient outcome. Clin. Microbiol. Infect..

[19] Lepe J.A., Guerrero F.J., Garrido A., Perea R. (2001). Deteccion de *Brucella melitensis* por el sistema BACTEC 9050. Enferm. Infecc. Microbiol. Clin..

[20] Fatolahzadeh B., Maleknejad P., Hejazi M.J., Pyri H. (2009). Development and evaluation of TUMS medium, a novel biphasic culture medium for isolation of *Brucella* spp. from patients. Iran. J. Microbiol..

[21] Yagupsky P., Peled N., Press J., Abu-Rashid M., Abramson O. (1997). Rapid detection of *Brucella melitensis* from blood cultures by a commercial system. Eur. J. Clin. Microbiol. Infect. Dis..

[22] Sagi M., Nesher L., Yagupsky P. (2017). The Bactec FX blood culture system detects *Brucella melitensis* bacteremia in adult patients within the routine 1-week incubation period. J. Clin. Microbiol..

[23] Akcam F.Z., Yayli G., Uskun E., Kaya O., Demir C. (2006). Evaluation of the Bactec microbial detection system for culturing miscellaneous sterile body fluids. Res. Microbiol..

[24] Cohen F.B., Robins B., Lipstein W. (1957). Isolation of *Brucella abortus* by percutaneous liver biopsy. N. Engl. J. Med..

[25] Fogel R., Lewis S. (1960). Diagnosis of *Brucella melitensis* by percutaneous needle biopsy of the liver. Ann. Intern. Med..

[26] Mantur B.G., Mulimani M.S., Bidari L.H., Akki A.S., Tikare N.V. (2008). Bacteremia is as unpredictable as clinical manifestations in human brucellosis. Int. J. Infect. Dis..

[27] Poston M.A., Parsons P.B. (1940). Isolation of *Brucella* from lymph nodes. J. Infect. Dis..

[28] Ruiz Castaneda M. (1961). Laboratory diagnosis of brucellosis in man. Bull. World Health Org..

[29] Young E.J. (1995). An overview of human brucellosis. Clin. Infect. Dis..

[30] Wang X., Yan Y., Wu F., Su G., Li S., Yuan X., Lai J., Zhou Z. (2018). Sixteen Chinese pediatric brucellosis patients onset of fever in non-epidemic areas and 8 developed with osteoarticular involvement. Clin. Rheumatol..

[31] Araj G.F. (2010). Update on laboratory diagnosis of human brucellosis. Int. J. Antimicrob. Agents.

[32] Diaz R., Casanova A., Ariza J., Moriyon I. (2011). The Rose Bengal Test in human brucellosis: A neglected test for the diagnosis of a neglected disease. PLoS Negl. Trop. Dis..

[33] Al Dahouk S., Nockler K. (2011). Implications of laboratory diagnosis on brucellosis therapy. Expert Rev. Anti Infect. Ther..

[34] Park S.H., Lee Y.H., Chu H., Hwang S.D., Hwang K.J., Choi H.Y., Park M.Y. (2012). Application of the microagglutination test for serologic diagnosis of human brucellosis. Osong Public Health Res. Perspect..

[35] Buchanan T.M., Faber L.V. (1980). 2-Mercaptoethanol *Brucella* agglutination test: Usefulness for predicting recovery from brucellosis. J. Clin. Microbiol..

[36] Pellicer T., Ariza J., Foz A., Pallares R., Gudiol F. (1988). Specific antibodies detected during relapse of human brucellosis. J. Infect. Dis..

[37] Borsa B.A., Aldag M.E., Yilmaz M., Dalar Z.G., Ozalp V.C. (2016). Comparison of a novel test (ODAK *Brucella* Coombs Gel Test) with commonly used serological tests in human brucellosis. Clin. Lab..

[38] Hanci H., Igan H., Uyanik M.H. (2017). Evaluation of a new and rapid serologic test for detecting brucellosis: *Brucella* Coombs gel test. Pak. J. Biol. Sci..

[39] Farrell I.D., Hinchliffe P.M., Robertson L. (1975). The use of the conglutinating complement fixation test in the diagnosis of human brucellosis. J. Hyg..

[40] Casanova A., Ariza J., Rubio M., Masuet C., Diaz R. (2009). BrucellaCapt versus classical tests in the serological diagnosis and management of human brucellosis. Clin. Vaccine Immunol..

[41] Orduna A., Almaraz A., Prado A., Gutierrez M.P., Garcia-Pascual A., Duenas A., Cuervo M., Abad R., Hernandez B., Lorenzo B. (2000). Evaluation of an immunocapture-agglutination test (BrucellaCapt) for serodiagnosis of human brucellosis. J. Clin. Microbiol..

[42] Pabuccuoglu O., Ecemis T., El S., Coskun A., Akcali S., Sanlidag T. (2011). Evaluation of serological tests for diagnoisis of brucellosis. Jpn. J. Infect. Dis..

[43] Araj G.F., Lulu A.R., Khateeb M.I., Saadah M.A., Shakir R.A. (1988). ELISA versus routine tests in the diagnosis of patients with systemic and neurobrucellosis. APMIS.

[44] McGiven J.A., Thompson I.J., Commander N.J., Stack J.A. (2009). Time resolved fluorescent resonance energy transfer assay for simple and rapid detection of anti-*Brucella* antibodies in ruminant serum sample. J. Clin. Microbiol..

[45] Lucero N.E., Escobar G.I., Ayala S.M., Silva P.P., Nielsen K. (2003). Fluorescence polarization assay for diagnosis of human brucellosis. J. Med. Microbiol..

[46] Li G., Rong Z., Wang S., Zhao H., Piao D., Yang X., Tian G., Jiang H. (2020). Rapid detection of brucellosis using a quantum dot-based immunochromatographic test strip. PLoS Negl. Trop. Dis..

[47] Matar G.M., Khneisser I.A., Abdelnoor A.M. (1996). Rapid laboratory confirmation of human brucellosis by PCR analysis of a target sequence on the 31-kilodalton *Brucella* antigen DNA. J. Clin. Microbiol..

[48] Navarro E., Escribano J., Fernandez J., Solera J. (2002). Comparison of three different PCR methods for detection of *Brucella* spp. in human blood samples. FEMS Immunol. Med. Microbiol..

[49] Zerva L., Bourantas K., Mitka S., Kansouzidou A., Legakis N.J. (2001). Serum is the preferred clinical specimen for diagnosis of human brucellosis by PCR. J. Clin. Microbiol..

[50] Li M., Zhou X., Li J., Sun L., Chen X., Wang P. (2018). Real-time PCR assays for diagnosing brucellar spondylitis using formalin-fixed paraffin embedded tissues. Medicine.

[51] Becker G.N., Tuon F.F. (2021). Comparative study of IS711 and bcsp31-based polymerase chain reaction (PCR) for the diagnosis of human brucellosis in whole blood and serum samples. J. Microbiol. Methods.

[52] Leal-Klevezas D.S., Martinez-Vazquez I.O., Lopez-Merino A., Martinez-Soriano J.P. (1995). Single-step PCR for detection of *Brucella* spp. From blood and milk of infected animals. J. Clin. Microbiol..

[53] Kattar M.M., Zalloua P.A., Araj G.F., Samaha-Kfoury J., Shbaklo H., Kanj S.S., Khalife S., Deeb M. (2007). Development and evaluation of real-time polymerase chain reaction assays on whole blood and paraffin embedded tissues for rapid diagnosis of human brucellosis. Diagn. Microbiol. Infect. Dis..

[54] Mitka S., Anetakis C., Souliou E., Diza E., Kansouzidou A. (2007). Evaluation of different PCR assays for early detection of acute and relapsing brucellosis in humans in comparison with conventional methods. J. Clin. Microbiol..

[55] Baddour M.M., Alkhalifa D.H. (2008). Evaluation of three polymerase chain reaction techniques for detection of *Brucella* DNA in peripheral human blood. Can. J. Microbiol..

[56] Baily G.G., Krahn J.B., Drasar B.S., Stoker N.G. (1992). Detection of *Brucella melitensis* and *Brucella abortus* by DNA amplification. J. Trop. Med. Hyg..

[57] Bounaadja L., Albert D., Chenais B., Henault S., Zygmunt M.S., Poliak S., Garin-Bastuji B. (2009). Real-time PCR for identification of *Brucella* spp.: A comparative study of IS711, *bcsp31*, and *per* target genes. Vet. Microbiol..

[58] Romero C., Gamazo C., Pardo M., Lopez-Goni I. (1995). Specific detection of *Brucella* DNA by PCR. J. Clin. Microbiol..

[59] Al-Nakkas A., Mustafa A.S., Wright S.G. (2005). Large-scale evaluation of a single-tube nested PCR for the laboratory diagnosis of human brucellosis in Kuwait. J. Med. Microbiol..

[60] Higuchi R., Fockler C., Dollinger G., Watson R. (1993). Kinetic PCR analysis: Real-time monitoring of DNA amplification reactions. Nat. Biotechnol..

[61] Navarro E., Serrano-Heras G., Castano M.J., Solera J. (2015). Real-time PCR detection chemistry. Clin. Chim. Acta..

[62] Sanjuan-Jimenez R., Colmenero J.D., Bermudez P., Alonso A., Morata P. (2013). Amplicon DNA melting analysis for the simultaneous detection of *Brucella* spp. and *Mycobacterium tuberculosis* complex. Potential use in rapid differential diagnosis between extrapulmonary tuberculosis and focal complications of brucellosis. PLoS ONE.

[63] Wittwer C.T., Herrmann M.G., Moss A.A., Rasmussen R.P. (1997). Continuous fluorescence monitoring of rapid cycle DNA amplification. Biotechniques.

[64] Zambardi G., Druetta A., Roure C., Fouque B., Girardo P., Chypre C., Marchand J., Freney J., Fleurette J. (1995). Rapid diagnosis of *Mycobacterium tuberculosis* infections by ELISA-like detection polymerase chain reaction products. Mol. Cell Probes.

[65] Moeini-Zanjani A., Pournajaf A., Ferdosi-Shahandashti E., Gholami M., Masjedian F., Khafri S., Rajabnia R. (2020). Comparison of loop-mediated isothermal amplification and conventional PCR tests for diagnosis of common *Brucella* species. BMC Res. Notes.

[66] Bricker B.J., Halling S.M. (1995). Enhancement of the *Brucella* AMOS PCR assay for differentiation of *Brucella abortus* vaccine strains S19 and RB51. J. Clin. Microbiol..

[67] Gopaul K.K., Sells J., Lee R., Beckstrom-Sternberg S.M., Foster J.T., Whatmore A.M. (2014). Development and assessment of multiplex high resolution melting assay as a tool for rapid single-tube identification of five *Brucella* species. BMC Res. Notes.

[68] Imaoka K., Kimura M., Suzuki M., Kamiyama T., Yamada A. (2007). Simultaneous detection of the genus *Brucella* by combinatorial PCR. Jpn. J. Infect. Dis..

[69] Lopez-Goni I., Garcia-Yoldi D., Marin C.M., de Miguel M.J., Munoz P.M., Blasco J.M., Jacques I., Grayon M., Cloeckaert A., Ferreira A.C. (2008). Evaluation of a multiplex PCR assay (Bruce-ladder) for molecular typing of all *Brucella* species, including the vaccine strains. J. Clin. Microbiol..

[70] Scholz H.C., Pfeffer M., Witte A., Neubauer H., Al Dahouk S., Wernery U., Tomaso H. (2008). Specific detection and differentiation of *Ochrobactrum anthropi*, *Ochrobactrum intermedium* and *Brucella* spp. by a multiprimer PCR that targets the recA gene. J. Med. Microbiol..

[71] Wellinghausen N., Nockler K., Sigge A., Bartel M., Essig A., Poppert S. (2006). Rapid detection of *Brucella* spp. in blood cultures by fluorescence in situ hybridization. J. Clin. Microbiol..

[72] Nimri L.F. (2003). Diagnosis of recent and relapsed cases of human brucellosis by PCR assay. BMC Infect. Dis..

[73] Surucuoglu S., El S., Ural S., Gazi H., Kurutepe S., Taskiran P., Yurtsever S.G. (2009). Evaluation of real-time PCR method for rapid diagnosis of brucellosis with different clinical manifestations. Pol. J. Microbiol..

[74] Dadar M., Shahali Y., Wareth G., Arora P. (2019). Molecular diagnosis of acute and chronic brucellosis in humans. Microbial Technology for the Welfare of Society. Microorganisms for Sustainability.

[75] Vila A., Pagella H., Vera Bello G., Vicente A. (2016). *Brucella suis* bacteremia misidentified as *Ochrobactrum anthropi* by the VITEK 2 system. J. Infect. Dev. Ctries..

[76] Lista F., Reubsaet F.A.G., De Santis R., Parchen R.R., de Jong A.L., Kieboom J., van der Laaken A.L., Voskamp-Visser I.A., Fillo S., Jansen H.J. (2011). Reliable identification at the species level of *Brucella* isolated with MALDI-TOF. BMC Microbiol..

[77] Karger A., Melzer F., Timke M., Bettin B., Kostrzewa M., Nockler K., Hohmann A., Tomaso H., Neubauer H., Al Dahouk S. (2013). Interlaboratory comparison of intact-cell matrix-assisted laser desorption ionization time of flight mass spectrometry results for identification and differentiation of *Brucella* spp. J. Clin. Microbiol..

[78] Mesureur J., Arend S., Celliere B., Courault P., Cotte-Pattat P.J., Totty H., Deol P., Mick V., Girard V., Touchberry J. (2018). A MALDI-TOF MS database with broad genus coverage for species-level identification of *Brucella*. PLoS Negl. Trop. Dis..

[79] Poonawala H., Marrs Conner T., Peaper D.R. (2018). The briefcase: Misidentification of *Brucella melitensis* as *Ochrobactrum anthropi* by matrix-assisted laser desorption ionization-time of flight mass spectrometry (MALDI-TOF MS). J. Clin. Microbiol..

[80] Yagupsky P., Baron E.J. (2005). Laboratory-exposures to brucellae and implications for bioterrorism. Emerg. Infect. Dis..

[81] Noviello S., Gallo R., Kelly M., Limberger R.J., DeAngelis K., Cain L., Wallace B., Dumas N. (2004). Laboratory-acquired brucellosis. Emerg. Infect. Dis..

[82] Centers for Disease Control, National Institutes of Health (2020). Biosafety in Microbiological and Biomedical Laboratories.

[83] Abo-Shehada M.N., Odeh J.S., Abu-Essud M., Abuharfeil N. (1996). Seroprevalence of brucellosis among high-risk people in northern Jordan. Int. J. Epidemiol..

[84] Ariza J., Pellicer T., Pallares R., Foz A., Gudiol F. (1992). Specific antibody profile in human brucellosis. Clin. Infect. Dis..

[85] Eldin C., Parola P., Raoult D. (2018). Limitations of diagnostic tests for bacterial infections. Med. Mal. Infect..

[86] McGiven J.A. (2013). New developments in the immunodiagnosis of brucellosis in livestock and wildlife. Rev. Sci. Tech..

[87] McGiven J., Howells L., Duncombe L., Stack J., Vijaya Ganesh N., Guiard J., Bundle D.R. (2015). Improved serodiagnosis of bovine brucellosis by novel synthetic oligosaccharide antigens representing the capping M epitope elements of *Brucella* O-polysaccharide. J. Clin. Microbiol..

[88] Patra K.P., Saito M., Atluri V.L., Rolan H.G., Young B., Kerrinnes T., Smits H., Ricaldi J.N., Gotuzzo E., Gilman R.H. (2014). A protein conjugate approach to develop a monoclonal antibody-based antigen detection test for the diagnosis of human brucellosis. PLoS Negl. Trop. Dis..

[89] Seco-Mediavilla P., Verger J.M., Grayon M., Cloeckaert A., Marin C.M., Zygmunt M.S., Fernandez-Lago L., Vizcaino N. (2003). Epitope mapping of the *Brucella melitensis* BP26 immunogenic protein: Usefulness for diagnosis of sheep brucellosis. Clin. Diagn. Lab. Immunol..

[90] Tiwari A.K., Kumar S., Pal V., Bhardwaj B., Rai G.P. (2011). Evaluation of recombinant 10 kDa immunodominant region of BP26 protein of *Brucella abortus* for specific diagnosis of bovine brucellosis. Clin. Vaccine Immunol..

[91] Buzgan T., Karahocagil M.K., Irmak H., Baran A.I., Karsen H., Evirgen O., Akdeniz H. (2010). Clinical manifestations and complications in 1028 cases of brucellosis: A retrospective evaluation and review of the literature. Int. J. Infect. Dis..

[92] Colmenero J.D., Reguera J.M., Martos F., Sanchez-De-Mora D., Delgado M., Causse M., Martin-Farfan A., Juarez C. (1996). Complications associated with *Brucella melitensis* infection: A study of 530 cases. Medicine.

[93] Memish Z., Mah M.W., Al Mahmoud S., Al Shaalan M., Khan M.Y. (2000). *Brucella* bacteraemia: Clinical and laboratory observations in 160 patients. J. Infect..

[94] Al Dahouk S., Neubauer H., Hensel A., Schoneberg I., Nockler K., Alpers K., Merzenich H., Stark K., Jansen A. (2007). Changing epidemiology of human brucellosis, Germany, 1962–2005. Emerg. Infect. Dis..

